# The structure of the hexameric atrazine chlorohydrolase AtzA

**DOI:** 10.1107/S1399004715000619

**Published:** 2015-02-26

**Authors:** T. S. Peat, J. Newman, S. Balotra, D. Lucent, A. C. Warden, C. Scott

**Affiliations:** a CSIRO Biomedical Manufacturing, Parkville, Australia; b Research School of Chemistry, Australian National University, Canberra, Australia; c CSIRO Land and Water Flagship, Black Mountain, Canberra, Australia; d Division of Engineering and Physics, Wilkes University, Wilkes-Barr, Pennsylvania, USA

**Keywords:** atrazine chlorohydrolase

## Abstract

Atrazine chlorohydrolase (AtzA) was discovered and purified in the early 1990s from soil that had been exposed to the widely used herbicide atrazine. It was subsequently found that this enzyme catalyzes the first and necessary step in the breakdown of atrazine by the soil organism *Pseudomonas* sp. strain ADP. Although it has taken 20 years, a crystal structure of the full hexameric form of AtzA has now been obtained. AtzA is less well adapted to its physiological role (*i.e.* atrazine dechlorination) than the alternative metal-dependent atrazine chlorohydrolase (TrzN), with a substrate-binding pocket that is under considerable strain and for which the substrate is a poor fit.

## Abstract 2

The structure of atrazine chlorohydrolase (AtzA) is presented and is used to reinterpret data from genetic, biochemical and evolutionary studies, providing insight into why this recently evolved enzyme appears to be poorly adapted for its physiological substrate compared with the alternative metal-dependent atrazine dechlorinase TrzN.

## 1. Introduction   

The last century saw the introduction of a host of anthropo­genic compounds, such as pesticides and antibiotics, into the environment (Russell *et al.*, 2011 ▶; Copley, 2000 ▶). This influx of new compounds has introduced a range of new selection pressures through factors such as toxicity and the availability of abundant potential nutrient sources (Copley, 2000 ▶; Russell *et al.*, 2011 ▶; Wackett, 2009 ▶). Nature has evolved mechanisms to cope with these new selection pressures, including the establishment of new metabolic pathways and enzymes. These new metabolic pathways and their associated enzymes are a tremendous resource for advancing our understanding of the mechanisms and constraints that underpin the evolutionary process, particularly with respect to the acquisition of new enzymatic functions.

**Figure 1:**
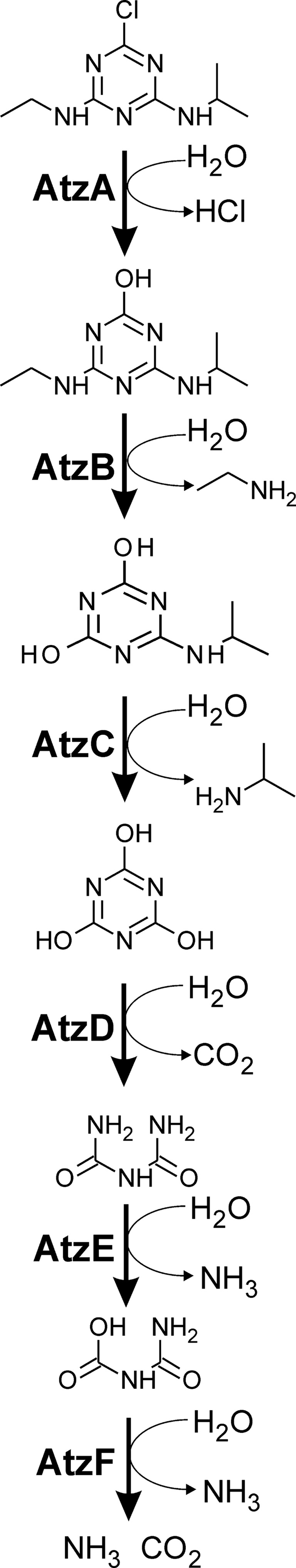
Atrazine catabolic pathway. The six hydrolytic steps of the atrazine catabolic pathway of *Pseudomonas* sp. strain ADP as catalysed by AtzA, AtzB, AtzC, AtzD, AtzE and AtzF are shown.

The bacterial atrazine catabolism pathway from *Pseudomonas* sp. strain ADP is a particularly well studied example of a metabolic pathway that has evolved in response to human perturbations of the chemical composition of the environment. The pathway is comprised of six enzymatic steps (Fig. 1 ▶): the sequential hydrolysis of the chloride and two *N*-alkyl chains to produce cyanuric acid by AtzA (de Souza *et al.*, 1996 ▶; Scott *et al.*, 2009 ▶), AtzB (Boundy-Mills *et al.*, 1997 ▶; Seffernick *et al.*, 2007 ▶) and AtzC (Sadowsky *et al.*, 1998 ▶; Shapir *et al.*, 2002 ▶), followed by a ring-opening hydrolysis (AtzD; Fruchey *et al.*, 2003 ▶; Seffernick *et al.*, 2012 ▶; Peat *et al.*, 2013 ▶) and two deamination steps (AtzE and AtzF; Balotra *et al.*, 2014 ▶, 2015 ▶; Shapir, Sadowsky *et al.*, 2005 ▶; Cameron *et al.*, 2011 ▶; Martinez *et al.*, 2001 ▶). The catabolism of cyanuric acid possibly predates the introduction of anthropogenic herbicides, as cyanuric acid is a naturally occurring compound (albeit not an abundant one; Wackett, 2009 ▶). However, the enzymes that catalyze the first three steps of the pathway (AtzA, AtzB and AtzC) are likely to have evolved recently in response to the presence of atrazine in the environment (Wackett, 2009 ▶; Udiković-Kolić *et al.*, 2013 ▶).

The atrazine chlorohydrolase AtzA has attracted a great deal of attention as a model for studying the evolutionary process, not least because of its relationship to melamine deaminase (TriA). AtzA and TriA share 98% identity, differing by just nine amino acids over their ∼470-amino-acid length, and each difference is conferred by one of just nine point mutations (Seffernick *et al.*, 2001 ▶; Noor *et al.*, 2012 ▶). However, AtzA and TriA have quite distinct enzymatic functions, with AtzA only capable of hydrolytic dehalogenation (Fig. 1 ▶) and the deaminase TriA possessing only low levels of promiscuous dehalogenase activity (Seffernick *et al.*, 2001 ▶). There have been studies that have used DNA shuffling (Raillard *et al.*, 2001 ▶) and evolutionary trajectory reconstruction (Noor *et al.*, 2012 ▶) to understand how these large functional differences could have evolved with so few genetic changes.

In other bacterial genera, particularly Gram-positive bacteria (*e.g.* *Arthrobacter* and *Nocardioides*), the role of AtzA is occupied by the alternative chlorohydrolase TrzN (Mulbry *et al.*, 2002 ▶; Sajjaphan *et al.*, 2004 ▶; Shapir *et al.*, 2006 ▶; Seffernick *et al.*, 2010 ▶). Both AtzA and TrzN belong to the same large family of amidohydrolases, although they are so different physically and phylogenetically that it is likely that atrazine chlorohydrolase activity evolved independently in each enzyme. While AtzA is a hexamer that contains one essential Fe^2+^ per monomer (de Souza *et al.*, 1996 ▶; Scott *et al.*, 2009 ▶), TrzN (which is ∼25% identical to AtzA) is a dimer that has a single Zn^2+^ bound in each active site (Shapir *et al.*, 2006 ▶). Moreover, while AtzA can only hydrolyze triazine halides, TrzN can hydrolyze a broad range of substituents (including halide, –OCH_3_ and –SCH_3_ groups) from both triazines and pyrimidines (Shapir, Rosendahl *et al.*, 2005 ▶; de Souza *et al.*, 1996 ▶). TrzN is also an order of magnitude more efficient than AtzA, with a *k*_cat_/*K*_m_ value of ∼10^5^ s^−1^ *M* ^−1^ compared with ∼1.5 × 10^4^ s^−1^ *M* ^−1^. This difference is in large part owing to the relatively high *K*_m_ of AtzA, which is greater than the water solubility of atrazine (153 µ*M*; Scott *et al.*, 2009 ▶), compared with that of TrzN for atrazine (∼20 µ*M*; Jackson *et al.*, 2014 ▶). In contrast to most other known hydrolytic dehalogenases, which use an active-site carboxylic acid (Asp) to displace the halide ion (Verschueren *et al.*, 1993 ▶; Newman *et al.*, 1999 ▶), the metal-dependent reaction mechanisms of AtzA and TrzN make these two enzyme lineages somewhat unusual in nature.

Despite intensive genetic and biochemical study of AtzA, the lack of an experimentally derived structural model has hampered efforts to understand the details of the sequence–function relationship. Here, we present the first X-ray structure of AtzA, upon which we have based a reinterpretation of the genetic and biochemical analyses of this model chloro­hydrolase.

## 2. Materials and methods   

### 2.1. Protein expression and purification   

The construction of the pCS150 expression vector containing the wild-type *atzA* gene has been described elsewhere (Scott *et al.*, 2009 ▶). The mutant *atzA* gene encoding the AtzA Ala170Thr, Met256Ile, Pro258Thr, Tyr261Ser variant was obtained from Life Technologies (Australia) and provided in pMK-RQ with NdeI and BamHI sites placed for subcloning into the pCS150 vector. pCS150 plasmids containing either the wild-type or mutant *atzA* genes were used to transform electrocompetent *Escherichia coli* BL21 λDE3 cells (Invitrogen) and were grown at 310 K on Luria–Bertani (LB; Lennox, 1955 ▶) agar [1.5%(*w*/*v*)] plates supplemented with 100 µg ml^−1^ ampicillin. Overnight starter cultures of 50 ml were inoculated with a single colony. The overnight cultures were diluted 1:20 into 950 ml LB medium and were shaken at 310 K and 200 rev min^−1^ until an OD_600_ of 0.6–0.8 was obtained. Protein expression was initiated by the addition of 100 µ*M* isopropyl β-d-1-thiogalactopyranoside (IPTG). The induced cultures were kept at 310 K overnight whilst shaking at 200 rev min^−1^.

The cells were then harvested by centrifugation at 4000*g* for 10 min in an Avanti J-E centrifuge (Beckman Coulter, Indianapolis, USA), resuspended in lysis buffer (50 m*M* HEPES, pH 7.5, 100 m*M* NaCl) and lysed by passage through an Avestin C3 homogenizer three times at 124 MPa. Insoluble cellular debris was removed by centrifugation at 21 000*g* using an Avanti J-E centrifuge.

AtzA and the AtzA variant were purified from lysed cells in three steps: His-tag affinity chromatography using an Ni–NTA Superflow Cartridge (Qiagen, Maryland, USA) with a gradient of 0–300 m*M* imidazole in 50 m*M* HEPES pH 7.5, 100 m*M* NaCl and size-exclusion chromatography using a 130 ml column packed with Superdex 200 prep-grade resin (GE Healthcare Life Sciences, Australia) with a buffer composed of 50 m*M* HEPES pH 7.5, 100 m*M* NaCl. The His tag was removed by thrombin proteolysis and the protein was then purified by size-exclusion chromatography with a Superdex 200 column. The protein was concentrated in an Amicon Ultra-15 Centrifugal Filter Unit with an Ultracel-30 membrane (Millipore, Carrigtwohill, Ireland) to 11.6 mg ml^−1^ and snap-frozen in liquid nitrogen in 100 µl aliquots. The final purity was estimated to be 98% from a Coomassie-stained gel and typical yields were 5–7 mg purified protein from 1 l of LB medium.

### 2.2. Crystallization and structure solution   

All crystallization experiments were performed in 96-well SD-2 plates (IDEX, USA) against a 50 µl reservoir. Initial crystals were obtained using droplets consisting of 150 nl concentrated protein solution (∼11 mg ml^−1^) combined with 150 nl reservoir solution and these were used in seeding experiments. The final crystals were obtained using droplets consisting of 150 nl concentrated protein solution combined with 120 nl reservoir solution and 30 nl seed stock. Either a Phoenix (ARI, USA) or a Mosquito (TTP Labtech, UK) robot was used to place the crystallization drops. Crystals (Supplementary Fig. S1) grew unreliably from a reservoir consisting of 5.5%(*w*/*v*) PEG 8000, 2.7%(*v*/*v*) diethylene glycol, 50 m*M* HEPES pH 7.1 at 281 K and were used to collect X-ray data on the MX-2 beamline of the Australian Synchrotron at a wavelength of 0.9537 Å (13 000 eV). The crystals were cryoprotected by the addition of reservoir solution supplemented with a further 20% diethylene glycol prior to cryocooling in liquid N_2_. Data were indexed with *XDS* (Kabsch, 2010 ▶) and scaled using *AIMLESS* (Evans, 2011 ▶). The structure was solved using molecular replacement (*Phaser*; McCoy *et al.*, 2007 ▶) with PDB entry 3hpa (32% sequence identity, 15 gaps; Hall *et al.*, 2010 ▶). A clear solution was only found when a dimer of 3hpa was used, with six dimers placed in the asymmetric unit. The space group was found to be *P*22_1_2_1_ and the resolution of the data extended to 2.8 Å (see Table 1 ▶). The model was initially built using *Buccaneer* (Cowtan, 2006 ▶) and was then rebuilt by hand using *Coot* (Emsley *et al.*, 2010 ▶) and refined using *REFMAC* (Murshudov *et al.*, 2011 ▶). Two full hexamers were found to be present in the asymmetric unit, each being a trimer of dimers. The model refined to *R*_work_ and *R*_free_ values of 19.3 and 22.2%, respectively (Table 1 ▶). The Ramachandran plot (from *Coot*) shows 94.9% of the residues in the most favourable region, 3.9% in the allowed region and 1.1% in the outlier region. After this original structure was built, a second set of crystals were obtained under similar conditions [50 m*M* HEPES pH 7.3, 4.6%(*w*/*v*) PEG 10 000] and another data set was obtained on the MX-2 beamline with data that extended to 2.2 Å resolution. This crystal was determined to belong to space group *P*2_1_2_1_2_1_ and a single hexamer was found in the asymmetric unit. The model and structure factors for both structures have been deposited in the PDB as entries 4v1x (2.2 Å resolution) and 4v1y (2.8 Å resolution).

### 2.3. Molecular modelling   

The gas-phase equilibrium conformation of the AtzA metal-coordination site was calculated using density functional theory with the B3LYP functional and the 6-31+G* basis set (with the LANL2DZ pseudopotential used for iron core electrons) as implemented in *Gaussian* 09 (Frisch *et al.*, 2009 ▶).

Atrazine was docked into a flexible AtzA binding pocket using the *RosettaLigand* docking protocol with the coordinates from PDB entry 4v1x. Partial atomic charges were calculated for the ligands using the AM1BCC Hamiltonian as implemented in *QuacPac* (v.1.6.3.1; OpenEye Scientific Software, Santa Fe, New Mexico, USA) and conformers were enumerated using *Omega* (v.2.5.1.4; OpenEye Scientific Software, Santa Fe, New Mexico, USA). 10 000 docking trajectories were performed using the *RosettaLigand* docking protocol (Davis *et al.*, 2009 ▶). The output ensembles were culled to retain the top 5% of structures according to the total energy (computed from the *Rosetta* energy function). From this smaller ensemble, the top 20 protein–ligand complexes (according to protein–ligand interaction energy) were considered for final examination.

### 2.4. Dynamic light scattering   

Concentrated AtzA (11.6 mg ml^−1^) was diluted by 50% in a serial fashion with 50 m*M* HEPES pH 7.5, 100 m*M* NaCl to obtain a range of concentrations from 11.6 to 0.09 mg ml^−1^. 20 µl volumes of each sample, in duplicate, were placed in a 384-well plate with blanks consisting of just buffer alone. The plate was placed in a DynaPro plate reader DLS machine (Wyatt Technology, Santa Barbara, California, USA) and 50 spectra were obtained at 297 K for each sample and averaged. The top three concentrations gave a radius of gyration of 5.9 nm, whereas the lowest concentration (0.09 mg ml^−1^) gave an average radius of 5.2–5.3 nm. Measuring the hexamer always gave a diameter of greater than 10 nm (10.5 to 13.0 mm, depending on the points chosen), whereas the longest dimension seen for a dimer was 8.3 nm.

## 3. Results and discussion   

### 3.1. The structure of hexameric AtzA   

Analysis with *PDBeFold* revealed that the closest structure in the PDB to AtzA was that used for molecular replacement, PDB entry 3hpa (r.m.s.d. of 1.6 Å; Hall *et al.*, 2010 ▶), which was also the structure with the greatest amino-acid sequence identity (32% over 411 residues). Another structural genomics target, PDB entry 3lnp (Kube *et al.*, 2013 ▶), is the next closest by structural homology, followed by two adenosine deaminases from *Xanthomonas campestris* (PDB entry 4dzh; New York Structural Genomics Research Consortium, unpublished work) and *Pseudomonas aeruginosa* (PDB entry 3pao; New York Structural Genomics Research Consortium, unpublished work). The TrzN structure (Seffernick *et al.*, 2010 ▶; PDB entry 3lsb), which has 26% sequence identity over 414 residues and an r.m.s.d. of 1.8 Å, is the fifth most similar structure to AtzA in the PDB. All of these molecules are classified as amidohydrolases, with 3hpa proposed to be an 8-oxoguanine deaminase. Although 3lnp has a highly homologous active site (four histidine residues and an aspartic acid residue), a calcium ion was modelled into the density instead of either Fe^2+^ or Zn^2+^; all of the other top hits on the list have either Fe^2+^ or (more commonly) Zn^2+^ in their active sites.

**Figure 2:**
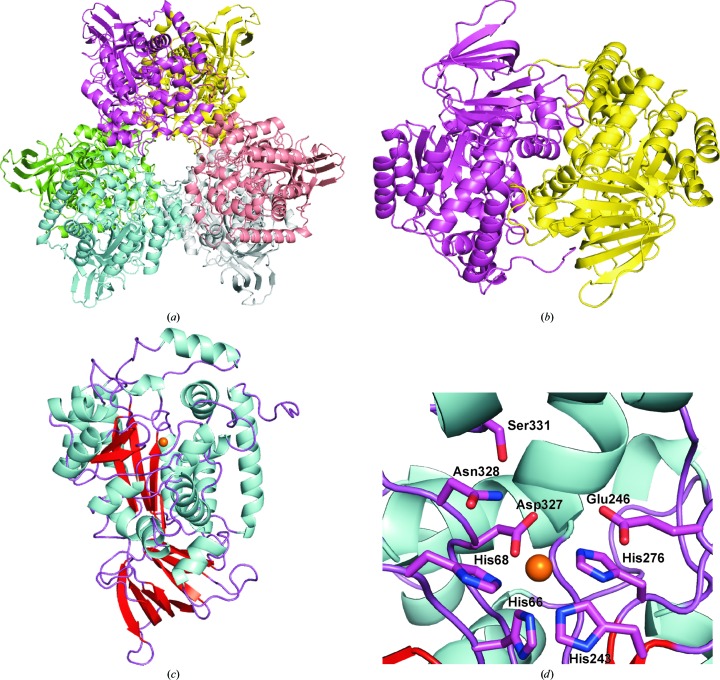
Structure of AtzA. (*a*) The X-ray structure of the AtzA hexamer is a trimer of dimers and is coloured by monomer. The structures of the dimer (*b*) (coloured by monomer), monomer (*c*) (coloured by secondary structure) and active site (*d*) are shown. The active-site metal (Fe^2+^) is shown as an orange sphere.

**Figure 3:**
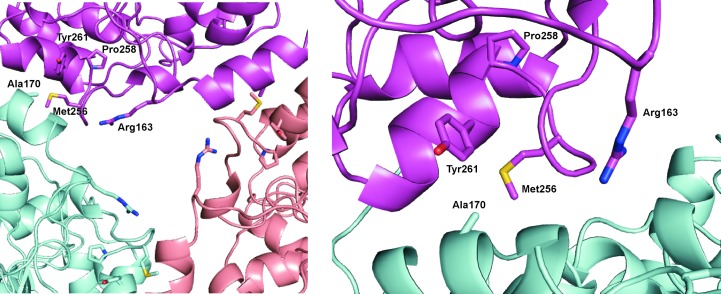
The entrance to the central cavity of the AtzA hexamer and the amino-acid residues of the hexamer-stabilizing interface. The entrance to the AtzA hexamer central cavity is largely formed by Arg163 contributed by each monomer. The hole formed in the hexamer is approximately 14 Å across at the entrance. Amino-acid substitutions at residues Ala170, Met256, Pro258 and Tyr261 destabilize the hexamer. The interactions between three monomers on one face of the hexamer (left) and at a single monomer–monomer interface (right) are shown.

The AtzA hexamer (about 315 kDa in molecular weight) is a trimer of dimers (Fig. 2 ▶ *a*), with the dimer interface being significantly larger than the individual protomer interfaces used to make up the hexamer (Figs. 2 ▶ *a* and 2 ▶ *b*): 3295 Å^2^ compared with 585 Å^2^. The hexamer was confirmed by DLS and size-exclusion chromatography (SEC; Supplementary Fig. S2), consistent with previous reports for the solution state of the protein (de Souza *et al.*, 1996 ▶; Scott *et al.*, 2009 ▶). The dimer surface covers 17–18% of the monomer (a single monomer has a surface area of ∼18 600 Å^2^; Fig. 2 ▶ *c*). The dimer interface is similar to that found in the other amidohydrolases and the dimer is similar enough in overall structure that the search model used for molecular replacement (PDB entry 3hpa) gave a significantly more robust solution when the dimer was used instead of the monomer. Two full hexamers (over 600 kDa) were found in the asymmetric unit of the crystal structure in space group *P*22_1_2_1_, whereas a single hexamer was found in space group *P*2_1_2_1_2_1_. The two structures are essentially identical (r.m.s.d. of 0.3 Å), with the higher resolution data giving greater clarity in amino-acid positions and having significantly more water molecules added to the model. The most obvious interaction seen when looking down the threefold axis of the hexamer is the 11-amino-acid insertion (relative to PDB entry 3hpa) that forms the loop and helix encompassing residues 160–183 that has not been seen in the other amidohydrolase structures to date. The ‘cavity’ in the hexamer is approximately 14 Å across at the entrance, with one residue in particular, Arg163, forming much of the surface of this entrance (Fig. 3 ▶).

The *atzA* genes from *Aminobacter aminovorans* isolates recently collected from vineyards in France (Noor *et al.*, 2014 ▶) have accumulated a variety of mutations that have been demonstrated to confer changes in substrate specificity and alter the *K*_d_ of the enzyme for Fe^2+^. Interestingly, four of the amino-acid changes conferred by these mutations (Ala170Thr, Met256Ile, Pro258Thr and Tyr261Ser) are located in the interface responsible for coordinating the AtzA hexamer (Fig. 3 ▶). AtzA variants carrying all four amino-acid substitutions were observed in the *A. aminovorans* isolates in the study by Noor *et al.* (2014 ▶). As these amino acids could effect a change in hexamer formation, the AtzA Ala170Thr, Met256Ile, Pro258Thr, Tyr261Ser variant was expressed and purified and its size was determined by SEC. Unlike the wild-type protein, which eluted from SEC with an estimated molecular weight of ∼300 kDa, the variant eluted with an estimated molecular weight of ∼100 kDa (*i.e.* a dimer; data not shown). This suggests that there is some factor that has selected dimeric AtzA in the more recent French isolates when compared with the AtzA from the original *Pseudomonas* isolate obtained from the USA, although it is uncertain whether the dimeric AtzA variants predate the hexameric form or *vice versa*.

### 3.2. Analysis of the AtzA active site   

**Figure 4:**
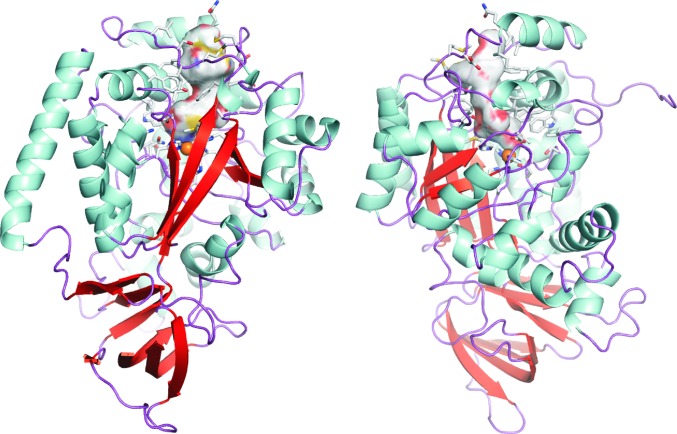
Access to the active site of AtzA. The surface of the active site and solvent-access channel (which form a single continuous channel from the active-site metal to the solvent) is shown in an AtzA monomer which has been coloured by secondary structure. The amino acids that form this surface (His66, His68, Gln71, Phe84, Tyr85, Trp87, Leu88, Phe89, Val92, Tyr93, Asp128, Met155, Phe157, Met160, Asp161, Ile164, Gln165, Val168, Leu180, Ser182, Ile183, Met184, Ala216, Thr217, Thr219, Ala220, His243, Glu246, Asp250, His276, Leu305, Asp327, Asn328 and Ser331) are shown as sticks, but have not been labelled for clarity. The amino-acid resides are labelled in Supplementary Fig. S5. The active-site metal (Fe^2+^) is shown as an orange sphere. The images are rotated 180° around the vertical axis with respect to each other.

The substrate-binding pocket and catalytic centre of each monomer is accessible *via* a long hydrophobic channel. The channel, substrate-binding pocket and active site are comprised of His66, His68, Gln71, Phe84, Tyr85, Trp87, Leu88, Phe89, Val92, Tyr93, Asp128, Met155, Phe157, Met160, Asp161, Ile164, Gln165, Val168, Leu180, Ser182, Ile183, Met184, Ala216, Thr217, Thr219, Ala220, His243, Glu246, Asp250, His276, Leu305, Asp327, Asn328 and Ser331 (Fig. 4 ▶). The identities of these residues are in good agreement with a homology model used to successfully predict sites for mutagenesis to substantially reduce the *K*_m_ for atrazine (*i.e.* Ala216, Thr217, Thr218, Ala219 and Asp250; Scott *et al.*, 2009 ▶).

The mononuclear active-site metal is coordinated by His66, His68, His243, His276 and Asp327 (Fig. 2 ▶ *d*); it is a subtype III amidohydrolase metal-binding motif similar to that found in *E. coli* adenine deaminase (Seibert & Raushel, 2005 ▶). The bond lengths between the bound cation and ligating amino-acid side chains are longer than would be expected for a metal centre of this type, with lengths in the following ranges: Fe–His66, 2.68–2.88 Å; Fe–His68, 2.76–2.96 Å; Fe–His243, 3.01–3.14 Å; Fe–His276, 2.76–2.99 Å; Fe–Asp327, 2.41–2.49 Å. Although the conformations of the metal-coordinating side chains found in the active site are indicative of an octahedral complex (as would be expected for Fe^2+^ binding), the geometry is distorted from that predicted by gas-phase electronic structure calculations (Supplementary Fig. S3). Iron-coordinating histidine residues at these distances are observed in other structures in the PDB, although they are far less frequent than those with shorter bond lengths (the mean His–Fe bond length is 2.2 Å, with a standard deviation of ∼0.2 Å; Supplementary Fig. S4). Although no negative density was seen when the Fe atoms were set to 100% occupancy, the resulting *B* factors were over double those for the nearby residues. As a result, we have set the occupancies of the Fe atoms to 50%, which gives much more reasonable *B* factors. These results imply that the Fe atom is bound but not tightly coordinated in the active site, consistent with the unusually high observed *K*_d_ of AtzA for iron (∼5 µ*M*; Noor *et al.*, 2014 ▶). Interestingly, Fe^2+^ bound in related amido­hydrolases (*e.g.* cytosine deaminases; PDB entries 1ra0, 1k70 and 4r88; Ireton *et al.*, 2001 ▶; Mahan *et al.*, 2004 ▶) is also found to be less tightly coordinated than Zn^2+^ cations, despite Fe^2+^ being the physiologically relevant cation.

**Figure 5:**
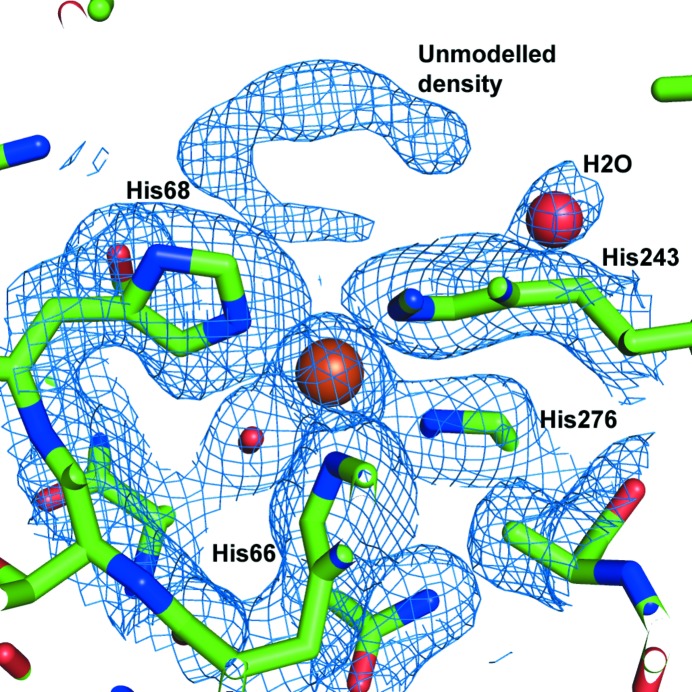
Density found in the active site of AtzA. A composite OMIT map was generated using the *CCP*4 package and the figure shows the ‘extra’ density seen in the active site which may be owing to atrazine (top of the figure, blue mesh). The active-site iron is shown as an orange sphere, water is shown as a red sphere and several active-site residues are shown with associated density. The OMIT map was set to 1σ.

There are a number of Ramachandran outliers associated with the active site, His66, His276, Glu298, Asp327 and Asn332, which all have good electron density (Fig. 5 ▶) and are unambiguously placed. His66, His276 and Asp327 bind the Fe^2+^ in the active site directly and introduce strain into the active site. Most of the other Ramachandran outliers are also in good electron density. The one exception is Glu251, which is in a region of weak electron density and is likely to be mobile in the protein. This mobile region sits over the substrate and may be mobile in order to allow substrate entry or exit. One other Ramachandran outlier, Thr368, is interesting as it is in the middle of a helix; the preceding residue, Ala367, which is also part of the helical structure, has its carbonyl hydrogen-bonded to another residue (Ile63) instead of being in line with the other carbonyls in the helix.

Examination of the binding pocket indicated that it might be difficult for atrazine to bind in an orientation that would permit nucleophilic substitution by an activated water (as is the case in TrzN). Atrazine was both soaked into crystals and co-crystallized with the protein, with the co-crystallization experiment resulting in crystals that diffracted to 2.2 Å resolution. However, only a small amount of broken density was found in the active site in some of the monomers of the co-crystals (Fig. 5 ▶). The density in the active site was insufficient to unambiguously place atrazine. For this reason, atrazine docking was investigated using extensive molecular-docking calculations with the *RosettaLigand* docking protocol (Davis *et al.*, 2009 ▶). The results of these calculations suggest that nonproductive binding modes are common for atrazine.

**Figure 6:**
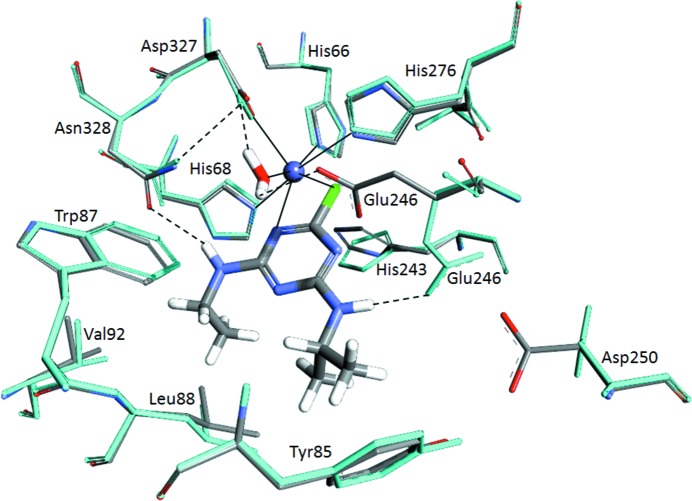
Simulated docking of atrazine in the AtzA active site: the highest scoring docked pose produced using the *RosettaLigand* docking protocol with the coordinates from PDB entry 4v1x that placed atrazine within a physically reasonable distance for nucleophilic substitution by the activated water. Although it is notionally possible to rotate the plane of the atrazine ring through 180°, the steric environment of the binding pocket appear to prohibit this alternative binding mode. The crystal structure (grey) and the modelled structure produced during docking simulations (cyan) are shown. The active-site metal (Fe^2+^) is shown as a blue sphere.

A high-scoring pose was produced that positioned the appropriate C atom of atrazine for nucleophilic attack by the metal-bound water. In contrast to our previously proposed mechanism based upon homology modelling (Scott *et al.*, 2009 ▶), the *N*-ethyl and *N*-iso­propyl moieties of the substrate are oriented away from the metal centre, making hydrophobic contacts with Val92, Trp87, Leu88, Tyr85 and Phe84 (Fig. 6 ▶). Phe84 presents a different rotamer to that found in the substrate-free crystal structure. The Cl atom and a ring N atom of atrazine are coordinated to the Fe^2+^, which positions the substrate ideally for nucleophilic attack by the coordinated nucleophile.

To determine whether the bidentate coordination mode is likely or whether there is monodentate coordination through the Cl atom, not requiring rotation of His243 but requiring a slight outwards movement of the hydrophobic pocket described above, would involve further investigation that is beyond the scope of the current work. Regardless, the lateral shift to form the monodentate coordination mode would be less than 1 Å and would be able to be accommodated without major reorganization of the active site.

There are two hydrogen-bonding interactions from the *N*-ethyl and *N*-isopropyl substituents to the side-chain O atom of Asn328 and the side chain of the new rotamer of Glu246, respectively. The latter of these residues may play a role in stabilizing the negative charge on a ring N atom during the formation of the tetrahedral intermediate. The symmetry of the triazine ring of the subunit could make it possible for the *N*-alkyl side chains of atrazine to bind in the opposite orientation to that presented here; however, the additional volume required to accommodate the *N*-isopropyl side chain compared with the *N*-ethyl side chain makes this alternate binding mode unlikely from a steric perspective.

The substrate-binding pocket of AtzA appears to be ill-adapted for binding atrazine, with strained conformers of key amino residues, a low-affinity metal-binding site and a high probability of unproductive substrate binding. These observations are consistent with the high *K*_m_ of wild-type AtzA for atrazine, which exceeds the aqueous solubility of the substrate (*i.e.* >159 µ*M*; Scott *et al.*, 2009 ▶), and may explain why we have been unable to capture clear X-ray structures of the AtzA–atrazine complex.

### 3.3. Location of the nine amino acids that distinguish AtzA and the melamine deaminase TriA   

**Figure 7:**
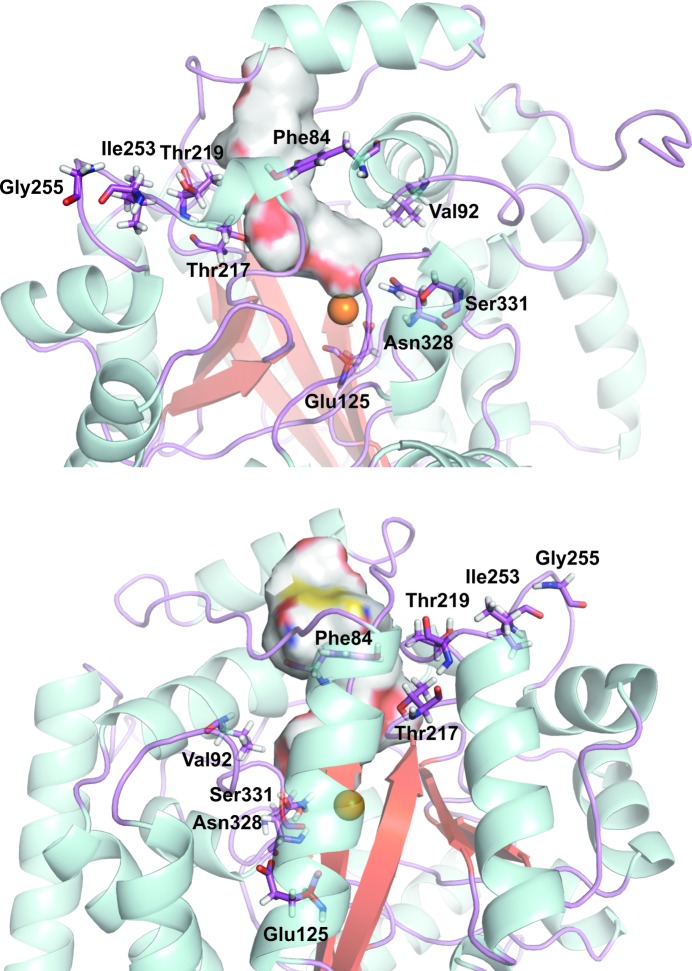
Differences between AtzA and the melamine deaminase TriA. The positions of Phe84, Val92, Asp125, Thr217, Thr219, Ile253, Gly255, Asn328 and Ser331, which distinguish AtzA from TriA, are shown in an AtzA monomer (coloured by secondary structure). The active-site metal (Fe^2+^) is shown as an orange sphere and the surface of the active site/solvent-access channel is shown. The images are rotated 180° around the vertical axis with respect to each other.

The melamine deaminase TriA differs from AtzA in the identities of just nine amino-acid residues: Leu84 (Phe), Leu92 (Val), Asp125 (Glu), Ile217 (Thr), Pro219 (Thr), Leu253 (Ile), Trp255 (Gly), Asp328 (Asn) and Cys331 (Ser) (AtzA residues shown in parentheses; Seffernick *et al.*, 2001 ▶). Of these nine amino-acid residues, six are part of the substrate-binding pocket/access channel in AtzA: Phe84, Val92, Thr217, Thr219, Asn328 and Ser331 (Fig. 7 ▶).

The presence of Phe84, Asn328 and Ser331 in the substrate-binding pocket is unsurprising, as these three amino acids had previously been shown to be the major determinants of atrazine/melamine specificity in AtzA and TriA, respectively (Noor *et al.*, 2012 ▶; Scott *et al.*, 2009 ▶; Raillard *et al.*, 2001 ▶). In particular, Ser331 and Asn328 are located within the vicinity of the active-site metal, which is consistent with their previously proposed role in catalysis in TriA-mediated melamine deamination and passive promotion of atrazine-mediated atrazine dechlorination (Noor *et al.*, 2012 ▶; Scott *et al.*, 2009 ▶).

In laboratory evolution experiments, altering Thr217 and Thr219 has been demonstrated to lead to substantial improvements in the *K*_m_ and *k*_cat_/*K*_m_ values of AtzA for atrazine (Scott *et al.*, 2009 ▶). Thr217 and Thr219 were targeted for mutagenesis as homology modelling had predicted their presence in the substrate-binding pocket, and the AtzA structure presented here confirms this (Fig. 7 ▶). Ile253 and Gly255 are not located in the substrate-binding pocket. Interestingly, however, Ile253 and Gly255 do cluster close to Thr219 (Fig. 7 ▶), with Ile253 within 4 Å of Thr219. Changes to the residues at positions 253 and 255 may therefore influence the packing of the residues of the substrate-binding pocket (*i.e.* positions 217 and 219), which is consistent with their relatively minor role in ‘tuning’ the specificity of the enzyme for melamine or atrazine (Noor *et al.*, 2012 ▶; Raillard *et al.*, 2001 ▶). The remaining difference between AtzA and TriA is at position 125 (Glu in AtzA and Asp in TriA) and has been shown to contribute substantially to the specificity of AtzA and TriA for dechlorination or deamination (Noor *et al.*, 2012 ▶). Although Glu125 is buried (Fig. 7 ▶), it does contribute to the active-site geometry *via* a hydrogen-bonding interaction between the Glu125 side chain and the backbone N atom of Val69, next to His68 which is bound to the Fe^2+^.

### 3.4. The catalytic mechanism of AtzA   

**Figure 8:**
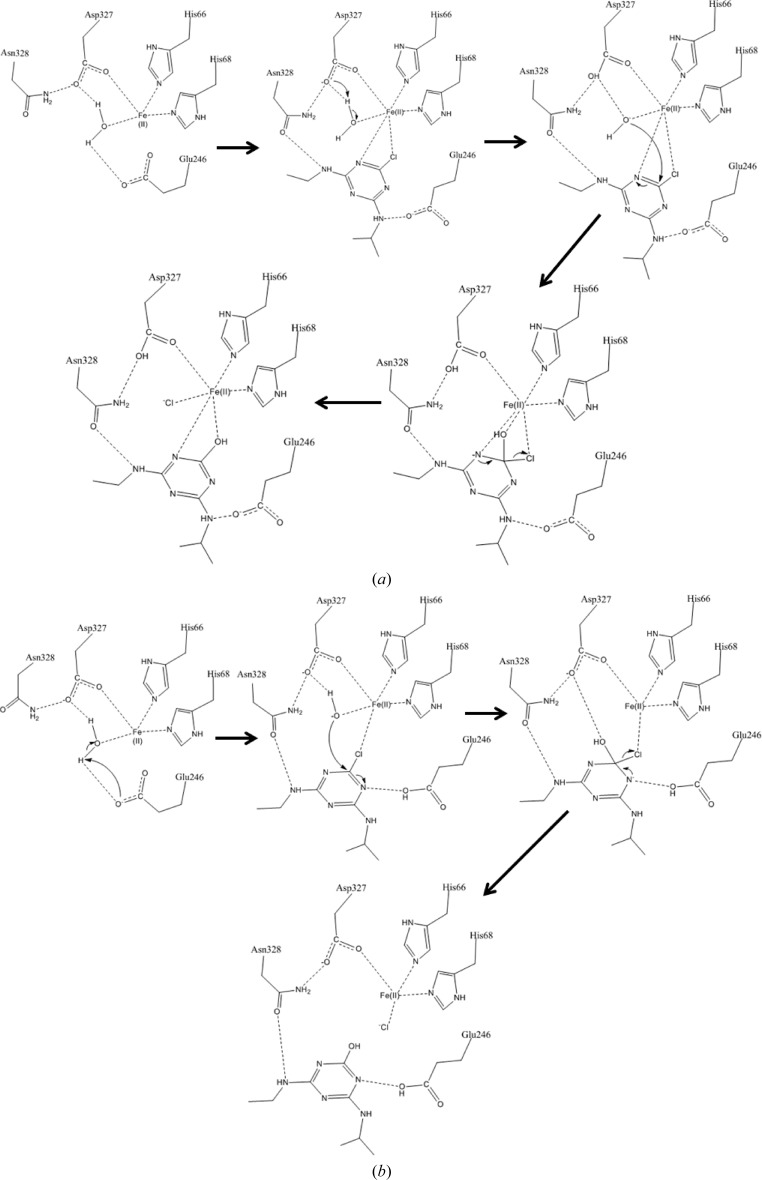
Plausible reaction mechanisms for AtzA. Two plausible reaction mechanisms are proposed involving either bidentate (*a*) or mondentate (*b*) coordination of the atrazine Cl atom to the Fe^2+^ centre of the AtzA active site.

We present two alternative potential catalytic mechanisms for the hydrolytic dechlorination of atrazine by AtzA that are constant with the *in silico* docking presented here, notwithstanding that empirical data will be required to test these and previously proposed reaction mechanisms. The first is based directly upon the interactions shown in the only docking pose that oriented atrazine appropriately for nucleophilic attack and the second is based upon the abovementioned monodentate coordination mode for atrazine (Figs. 8 ▶ *a* and 8 ▶ *b*, respectively) that better enables Glu246 to stabilize negative charge on another of the ring N atoms. In both of these mechanisms, coordination of the Cl atom to Fe^2+^ draws electron density from the C—Cl bond, increasing the susceptibility of the C atom to nucleophilic attack. In the first mechanism, the coordinated water is deprotonated by Asp327 followed by nucleophilic attack of the resulting activated hydroxide at the chlorine-bearing ring C atom. The negative charge of the tetrahedral intermediate is stabilized on the Fe^2+^-coordinated aromatic N atom, after which chloride is released and aromaticity is re-established in the hydroxylated product.

The second mechanism with monodentate atrazine coordination to Fe^2+^ involves deprotonation of the coordinated water by Glu246, which then rotates to donate a hydrogen bond to a ring N atom of atrazine upon substrate binding. This N atom then gains the negative charge of the tetrahedral intermediate, stabilized by the hydrogen bond from the protonated Glu246, followed by generation of the products as per the first mechanism.

It is possible that the ‘loose’ coordination geometry of the Fe^2+^ has evolved to facilitate the bidentate coordination of atrazine, which would not be possible with four ideally co­ordinating residues and a coordinated water. His243 in particular, at over 3 Å from the Fe^2+^, provides sufficient space for bidentate coordination, albeit by adopting a higher energy conformation. We note, however, that such a binding mode is unprecedented, and without a crystal structure of the AtzA–atrazine complex there is no empirical evidence to support it.

While the mechanisms for atrazine dechlorination that we have proposed, in light of the crystal structure and docking, differ from those that we have previously reported based upon homology modelling and manual placement of the substrate, the newly proposed mechanisms remain consistent with the empirically determined biochemical and mutagenesis data reported elsewhere (de Souza *et al.*, 1996 ▶; Raillard *et al.*, 2001 ▶; Scott *et al.*, 2009 ▶; Noor *et al.*, 2012 ▶). The sequential mutations reported earlier (Scott *et al.*, 2009 ▶) that reduce the atrazine dechlorination activity and increase the deaminase activity may do so through combinations of adjusted positioning of the metal-bound water through shifted hydrogen-bond networks, altering its p*K*_a_, and a changed steric factor in substrate binding and product release.

### 3.5. A comparison of the two metal-dependent atrazine chlorohydrolases AtzA and TrzN   

**Figure 9:**
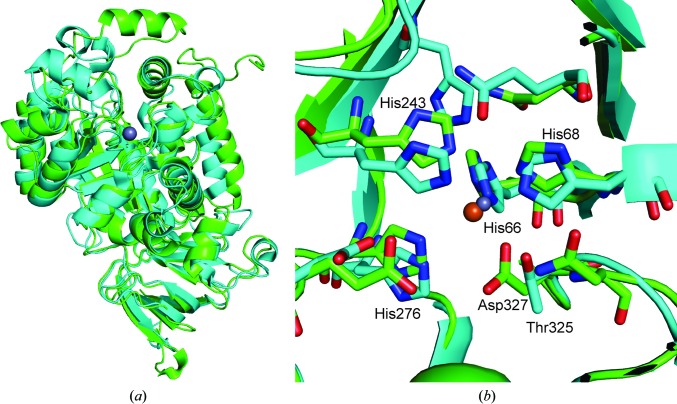
Comparison of AtzA with the alternative atrazine chlorohydrolase TrzN. (*a*) Superposition of an AtzA monomer (green) and a TrzN monomer (cyan). (*b*) Superposition of the active sites of AtzA (green) and a TrzN monomer (cyan). The active-site metals (Fe^2+^ and Zn^2+^; shown in orange and grey, respectively) are shown and the AtzA metal-binding ligands are labelled in green. The unique threonine (Thr325) of TrzN, which is equivalent to Asp327 in AtzA, is also labelled in cyan.

The tertiary structure of AtzA is similar to that of TrzN (Seffernick *et al.*, 2010 ▶). The monomers of AtzA and TrzN superpose with a root-mean-square difference of 1.8 Å on C^α^ atoms over 414 residues (Fig. 9 ▶ *a*). The metal-binding histidine side chains and metal overlay (Fig. 9 ▶ *b*), with the major differences being the longer metal–histidine distances (His66, His68 and His243 in AtzA at 2.7–3.1 Å instead of 2.0–2.1 Å in TrzN; Fig. 9 ▶ *b*), Fe^2+^ instead of Zn^2+^ as the metal, the additional His276 interaction with Fe^2+^ and the additional Fe–Asp327 interaction (at 2.4–2.5 Å). In the TrzN structure, the histidine equivalent to His276 (His274) makes a 2.7 Å hydrogen bond to the water coordinated to the Zn ion. Uniquely among the amidohydrolase family, the TrzN structure has a threonine residue at position 325, making it the first member of the ninth subgroup of metal-coordination sites of the amidohydrolase family (Seibert & Raushel, 2005 ▶; Jackson *et al.*, 2014 ▶; Khurana *et al.*, 2009 ▶).

Unlike the active site of AtzA, the TrzN active site has excellent complementarity for its substrate, which almost certainly accounts for the lower *K*_m_ of TrzN for atrazine (∼20 µ*M*; Shapir *et al.*, 2002 ▶) when compared with that of AtzA for the same substrate (>159 µ*M*). The mechanism of TrzN differs from any proposed for AtzA, in which a zinc-activated water forms the tetrahedral intermediate and highly activated leaving groups (such as halides) depart readily. Leaving groups with higher p*K*_a_ values (such as amines) can be hydrolyzed by TrzN as they are protonated; Glu241 first donates a proton to the triazine ring, which then protonates the leaving group. Thus, TrzN is an efficient hydrolase with a broad substrate range, while AtzA is a less active enzyme that can perform either deamination or dehalogenation reactions depending on the identities of the amino-acid residues at positions 328 and 331. AtzA variants that are effectively intermediates between AtzA and TriA possess both of these activities at the same time, but with a lower specificity than either AtzA or TriA (Noor *et al.*, 2012 ▶).

## 4. Concluding remarks   

For the first time since its discovery ∼20 years ago, we have been able to determine the structure of AtzA, the ‘archetypal’ atrazine chlorohydrolase. AtzA appears to be structurally ill-adapted to perform its physiological function, with a metal-coordination geometry that leads to a low affinity for its metal cofactor and a substrate-binding pocket that appears to be unable to accept its physiological substrate without substantial reorganization. In addition, four of the active-site amino-acid residues appear to be held in strained conformations, suggesting that the active site is far from optimized, which is consistent with the relatively poor kinetic performance of AtzA (*k*_cat_/*K*_m_ of ∼1 × 10^4^ *M* ^−1^ s^−1^ and a *K*_m_ in excess of the substrate’s solubility limit of 153 µ*M*; Scott *et al.*, 2009 ▶; Noor *et al.*, 2012 ▶). AtzA is certainly less well adapted to its physiological function than the alternative atrazine chlorohydrolase TrzN (*k*_cat_/*K*_m_ of ∼1 × 10^5^ *M* ^−1^ s^−1^ and a *K*_m_ value of ∼20 µ*M*; Jackson *et al.*, 2014 ▶), which has good complementarity for its substrate and a catalytic mechanism that permits a broader substrate range than AtzA. Both *atzA* and *trzN* are prone to horizontal gene transfer, as they are located on transmissible plasmids (de Souza *et al.*, 1998 ▶; Sajjaphan *et al.*, 2004 ▶), and it will be interesting to see whether the catalytically superior TrzN begins to outcompete and replace AtzA in the environment.

## Supplementary Material

PDB reference: AtzA, 4v1x

PDB reference: 4v1y

Supplementary Figures S1-S5.. DOI: 10.1107/S1399004715000619/rr5091sup1.pdf

## Figures and Tables

**Table 1:** Data-collection and refinement statistics Values in parentheses are for the highest resolution shell.

PDB code	4v1y	4v1x
Space group	*P*22_1_2_1_	*P*2_1_2_1_2_1_
Unit-cell parameters (, )	*a* = 117.5, *b* = 195.6, *c* = 283.9, = = = 90.0	*a* = 117.3, *b* = 146.1, *c* = 196.4, = = = 90.0
Resolution ()	49.52.80 (2.852.80)	48.72.20 (2.242.20)
*R*_merge_	0.229 (0.906)	0.235 (1.151)
*R*_meas_	0.266 (1.042)	0.249 (1.223)
*R*_p.i.m._	0.135 (0.531)	0.090 (0.438)
CC_1/2_	0.989 (0.795)	0.995 (0.840)
*I*/(*I*)	8.8 (2.6)	11.6 (3.2)
Completeness (%)	100 (100)	99.6 (99.2)
Multiplicity	7.5 (7.6)	15.1 (15.3)
Refinment		
Resolution ()	49.52.8	48.72.2
Unique reflections	153180	170140
*R*_work_/*R*_free_ (%)	19.3/22.2	16.6/19.6
No. of atoms		
Total	44810	23156
Protein	44565	22221
Other	105	42
Ions	15	6
Water	126	887
*B* factors (^2^)		
Overall	29.2	20.1
Protein	29.4	19.7
Other	32.4	36.7
Ions	29.2	24.3
Water	11.8	22.8
R.m.s. deviations		
Bond lengths ()	0.014	0.017
Bond angles ()	1.613	1.675
