# Supplementary material for: The structure of the hexameric atrazine chlorohydrolase AtzA
Source: Acta Crystallogr D Biol Crystallogr. 2015 Feb 26;71(Pt 3):710–20. doi: 10.1107/S1399004715000619 (PMC4356373; doi:10.1107/S1399004715000619)
Supplement: Supplementary file 1 [file d-71-00710-sup1.pdf]

BIOLOGICAL
CRYSTALLOGRAPHY

Volume 71 (2015)

Supporting information for article:

The structure of the hexameric atrazine chlorohydrolase AtzA

T. S. Peat, J. Newman, S. Balotra, D. Lucent, A. C. Warden and C. Scott

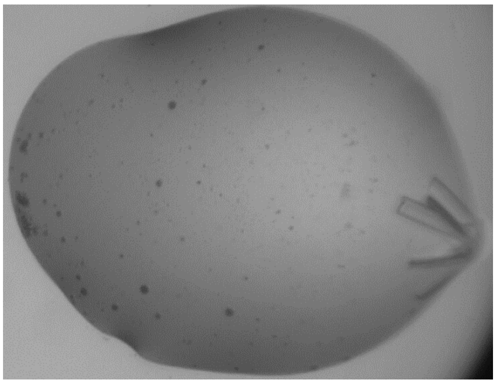

Supplementary Figure S1. AtzA Crystal.

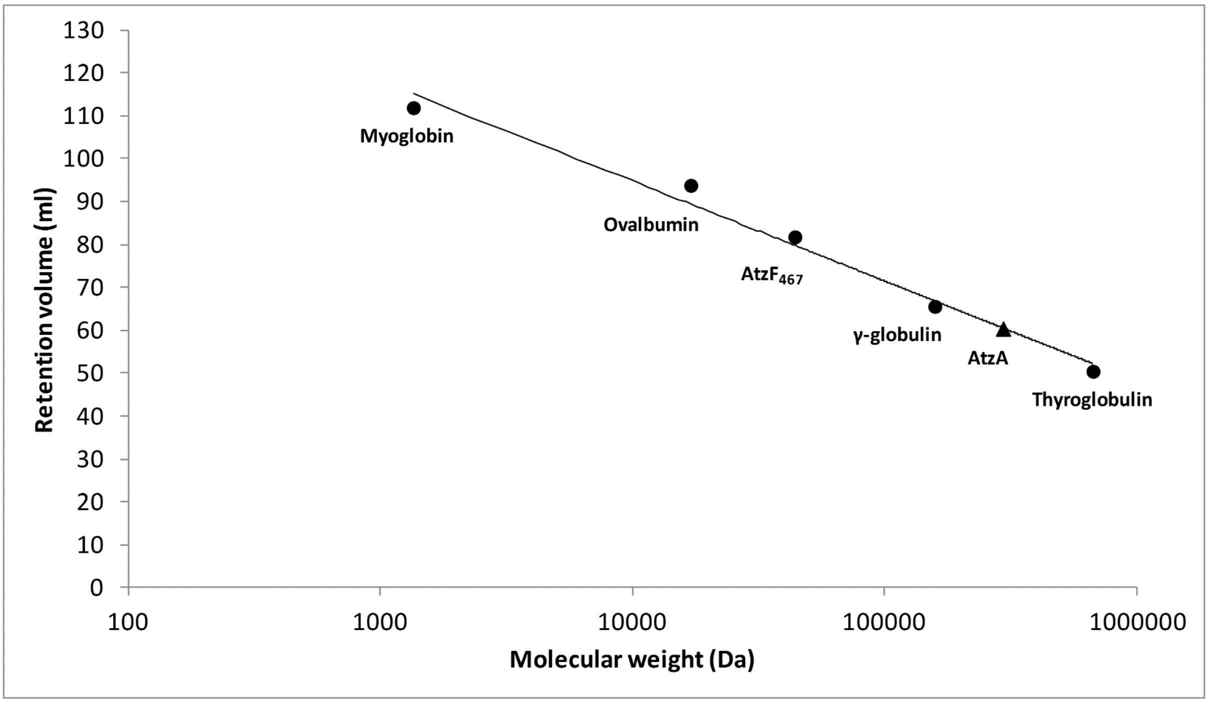

Supplementary Figure S2. Size exclusion chromatography (SEC) the AtzA hexamer.

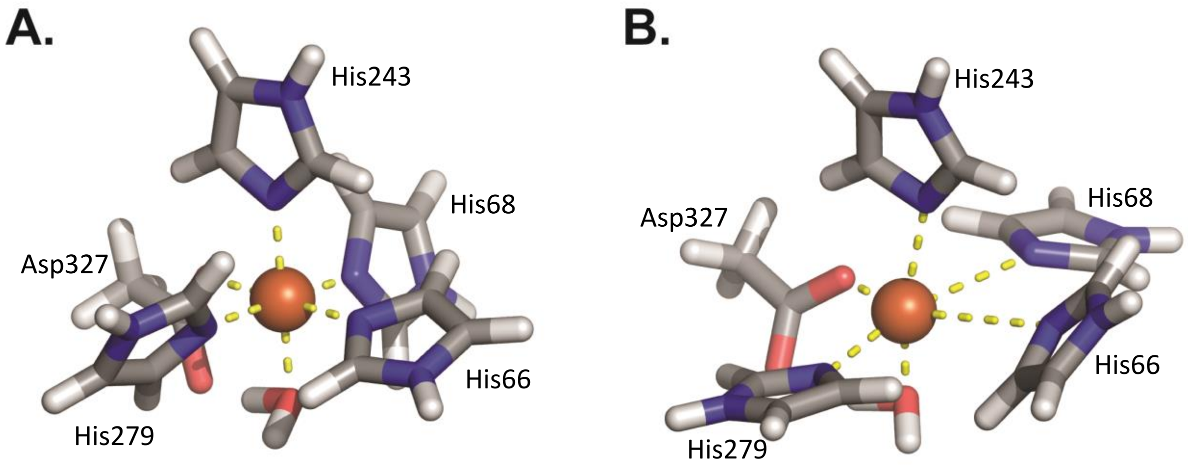

Supplementary Figure S3. The coordination geometry of the Fe^{2+} in AtzA predicted by gas-phase electronic structure calculation (A) and from the crystal structure (B).

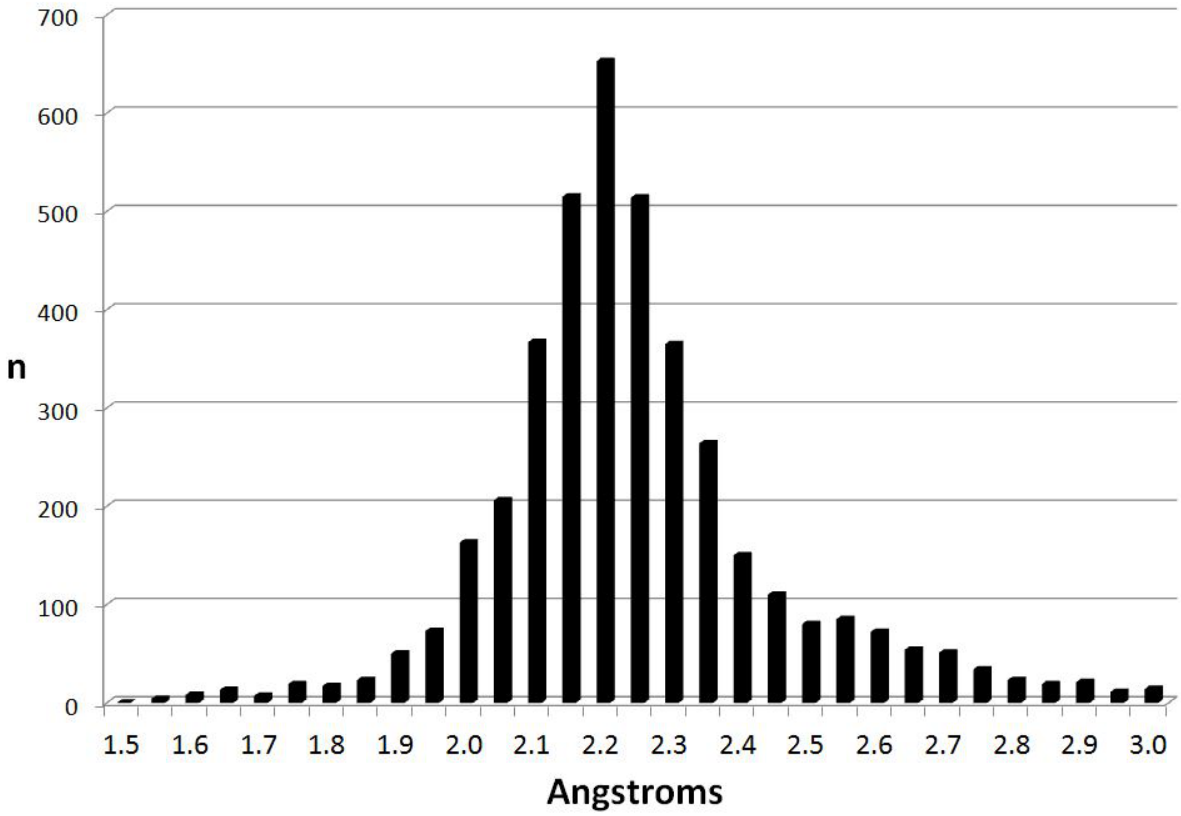

Supplementary Figure S4. Histogram showing the distribution of coordination bond lengths (< 3 Angstroms) between Fe^{2+} and the coordinating nitrogen in histidines in 517 structures in the Brookhaven Protein Databank.

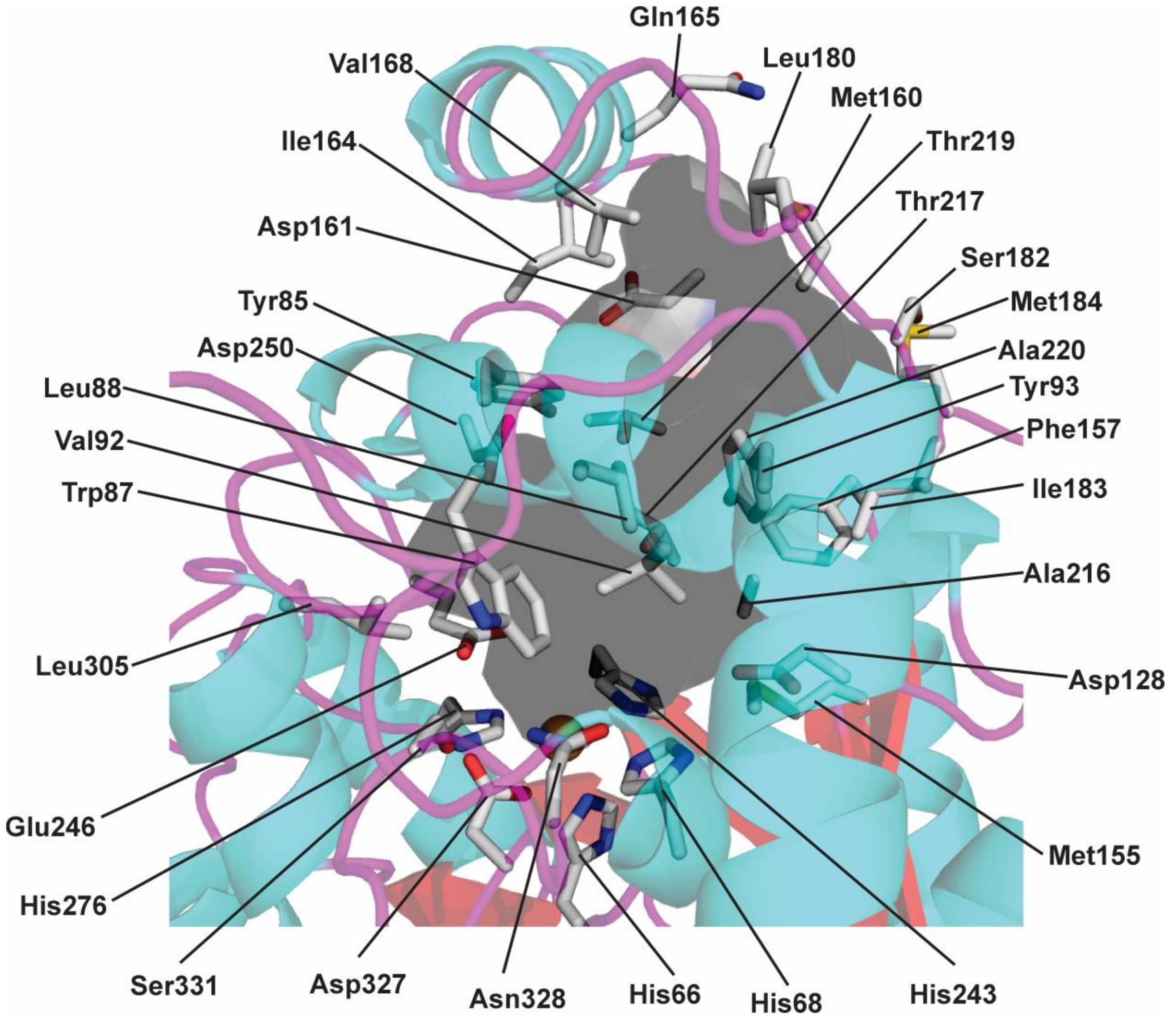

Supplementary Figure S5. The surface of the active site and solvent access channel is shown in an AtzA monomer, which has been colored by secondary structure. The amino acids that form this surface (His66, His68, Gln71, Phe84, Tyr85, Trp87, Leu88, Phe89, Val92, Tyr93, Asp128, Met155, Phe157, Met160, Asp161, Ile164, Gln165, Val168, Leu180, Ser182, Ile183, Met184, Ala216, Thr217, Thr219, Ala220, His243, Glu246, Asp250, His276, Leu305, Asp327, Asn328 and Ser331) are shown as sticks, and are labelled. The active-site metal (Fe^{2+}) is shown as an orange sphere.
